# High-speed optical tracking and augmented reality platform for image-guided interventions

**DOI:** 10.1117/1.JMI.13.3.035001

**Published:** 2026-06-16

**Authors:** Nati Nawawithan, James Yu, Kelden Pruitt, Baowei Fei

**Affiliations:** a University of Texas at Dallas, Center for Imaging and Surgical Innovation, Richardson, Texas, United States; b University of Texas at Dallas, Department of Bioengineering, Richardson, Texas, United States; c University of Texas Southwestern Medical Center, Department of Radiology, Dallas, Texas, United States

**Keywords:** augmented reality, optical tracking, image-guided interventions, laparoscopic surgery, biopsy, prostate

## Abstract

**Purpose:**

During interventional procedures, clinicians need to mentally register anatomical information from preoperative cross-sectional images onto the patient’s body to envision the location of subsurface targets and critical structures. This study aims to address this challenge by developing an augmented reality (AR)-based tracking system that can leverage cross-sectional images to provide real-time three-dimensional (3D) visualization of lesion targets and enable precise surgical procedures.

**Approach:**

Our AR platform combines a customized high-speed, real-time, optical tracking system, a holographic display device, a computer workstation, and a graphical user interface. The system displays holograms of virtual models of the target organ and surgical tools, as well as the navigation path. To validate our AR platform, we measured target registration errors (TRE) across two different types of interventional procedures using our high-speed optical tracking system. To conduct these experiments, we performed laparoscopic procedures and prostate biopsies on customized phantoms.

**Results:**

The integrated high-speed tracking and AR platform was applied to laparoscopic and biopsy procedures. The average TRE was 4.17±1.63  mm for laparoscopic procedures and 2.89±0.84  mm for prostate biopsy.

**Conclusions:**

An augmented reality platform with a high-speed precision optical tracking system was developed for interventional procedures. The AR platform has been demonstrated for potential applications in prostate laparoscopic and biopsy procedures and can be expanded to other interventional procedures.

## 1 Introduction

Medical imaging plays an important role in pre-operative planning, intra-operative guidance, and post-operative assessment.^1^^,^^2^ Clinicians use anatomical information acquired from various imaging modalities, such as computed tomography (CT) and magnetic resonance imaging (MRI), to localize lesions and define their relationship to adjacent organ structures. Conventionally, surgeons rely on mental mapping of preoperative anatomical information during surgery. However, this poses a significant challenge when lesions are located beneath the surface or obscured by complex anatomy.^3^

Augmented reality (AR) overlays virtual information on the real-world environment and can assist proceduralists by superimposing preoperative information on intraoperative images. This has potential to improve the safety and efficacy of interventional procedures.^4^^,^^5^ Detailed anatomical data can be relayed using virtual models, including the location of lesions deep in the organ, neurovascular bundles, and other hidden structures.^6^^,^^7^ AR can have various applications in interventional settings, due to these advantages.

Prostate cancer affects more than 300,000 men in the United States in 2025.^8^ Potential applications of AR-guided interventions in this field include laparoscopic prostatectomy and prostate biopsies, especially as intra-prostatic lesions are difficult to visualize on intra-operative ultrasound but are well-delineated on pre-operative MRI scans.^9^^,^^10^ The current definitive diagnosis for prostate cancer is biopsy, which is typically performed with ultrasound-guided transrectal and transperineal biopsy.^11^^–^^13^ AR guidance for prostate biopsy has been explored recently. For example, Majek et al.^14^ and Sparwasser et al.,^15^ respectively, utilized a head mounted display (HMD) and smart glasses to display virtual overlays, which assist targeted prostate biopsy by enhancing prostate detection rate. Our group investigated AR-guided systems for renal, prostate, and soft-tissue biopsy applications.^16^^–^^19^ Targeting accuracy at the millimeter level is essential for prostate biopsy, but the built-in depth sensor of current AR headsets can have a drift error at the centimeter level, therefore, they cannot be directly used for medical interventional procedures.

In addition, minimally invasive surgery (MIS) is another potential application for AR-guided systems. AR can enhance the surgeon’s view of the intra-abdominal cavity when operating through small incisions, which inherently limit the field of view and depth perception.^20^ A number of studies have shown that AR guidance can help in different MIS settings. Collins et al.^3^ created an AR pipeline demonstrating reliable registration accuracy in laparoscopic hysterectomy and uterine fibroid resection. Additional studies have shown that AR systems can assist with preoperative planning, decrease operative times, and improve clinical outcomes, such as shortening the length of hospital stay, for laparoscopic nephrectomy and hepatectomy.^21^^,^^22^ Moreover, AR technology can be integrated into robotic surgery systems. Porpiglia et al.^23^ achieved a registration error of less than 3 mm for robot-assisted radical prostatectomy. Although progress toward AR-guided surgical systems is ongoing, several challenges must be addressed before AR technology can be adopted in clinical settings. This includes real-time tracking of surgical tools and organ motion, improved hologram stability, shorter setup times, and user-specific eye calibration of the HMD.^4^^,^^5^

In this work, we integrate a customized, high-speed optical tracking system into our AR platform for prostate interventions. The platform enables real-time tracking of both surgical instruments and organ positions. These tracking data are utilized to register the physical anatomy with corresponding virtual models on the HMD. Finally, we evaluated the platform by measuring target registration errors using phantom models for prostate biopsy and laparoscopic procedures.

## 2 Methods

**Fig. 1:**
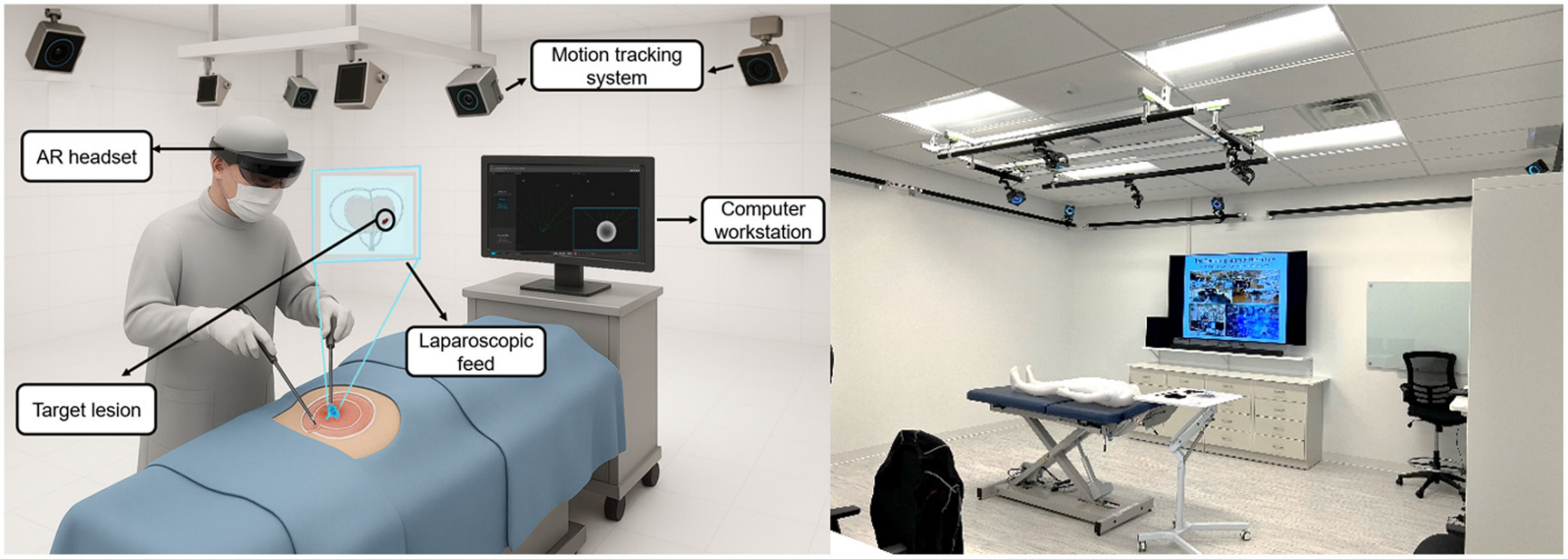
Overview of the high-speed optical tracking and AR-guided system. Left: The system comprises an AR headset for visualizing overlaid 3D models of the target lesion in real-time feed, and an optical tracking system for real-time tracking of the laparoscope, surgical tools, and the target organ through optical markers. Right: The laboratory setup for the AR-guided experiments.

Our AR-guided platform consists of two major components including an AR headset and a customized optical tracking system, as shown in Fig. 1. Unity (Unity Technologies, San Francisco, California, United States) was utilized as an AR development platform. Two surgical instruments, a 0 deg, 10 mm laparoscope (Olympus Surgical Technologies America, Bartlett, Tennessee, United States), and an optical tracking probe kit, serving as a simulated biopsy needle, were used to evaluate the AR platform.

The AR headset and the optical tracking system operate in different coordinate systems by default, requiring spatial alignment to accurately register the virtual model within the physical space. Two alignment approaches, QR code–based and transformation matrix–based, were investigated in this study. In the QR code method, the code position was defined as the origin of the optical tracking system. When the AR headset detects the QR code in the environment, the Microsoft Mixed Reality Toolkit (MRTK)^24^ automatically transforms the hologram’s position according to the QR code’s pose. In the transformation matrix method, three virtual dots corresponding to the retroreflective markers on the optical tracking probe were created. The user aligns these virtual dots with their respective physical markers on the probe while wearing the AR headset. Upon pressing the “Calibrate” button in our AR application, the transformation matrix is computed and applied to overlay the virtual model of the probe onto its real counterpart.

### 2.1 AR-Guided System for Laparoscopic Procedure

#### 2.1.1 System overview

**Fig. 2:**
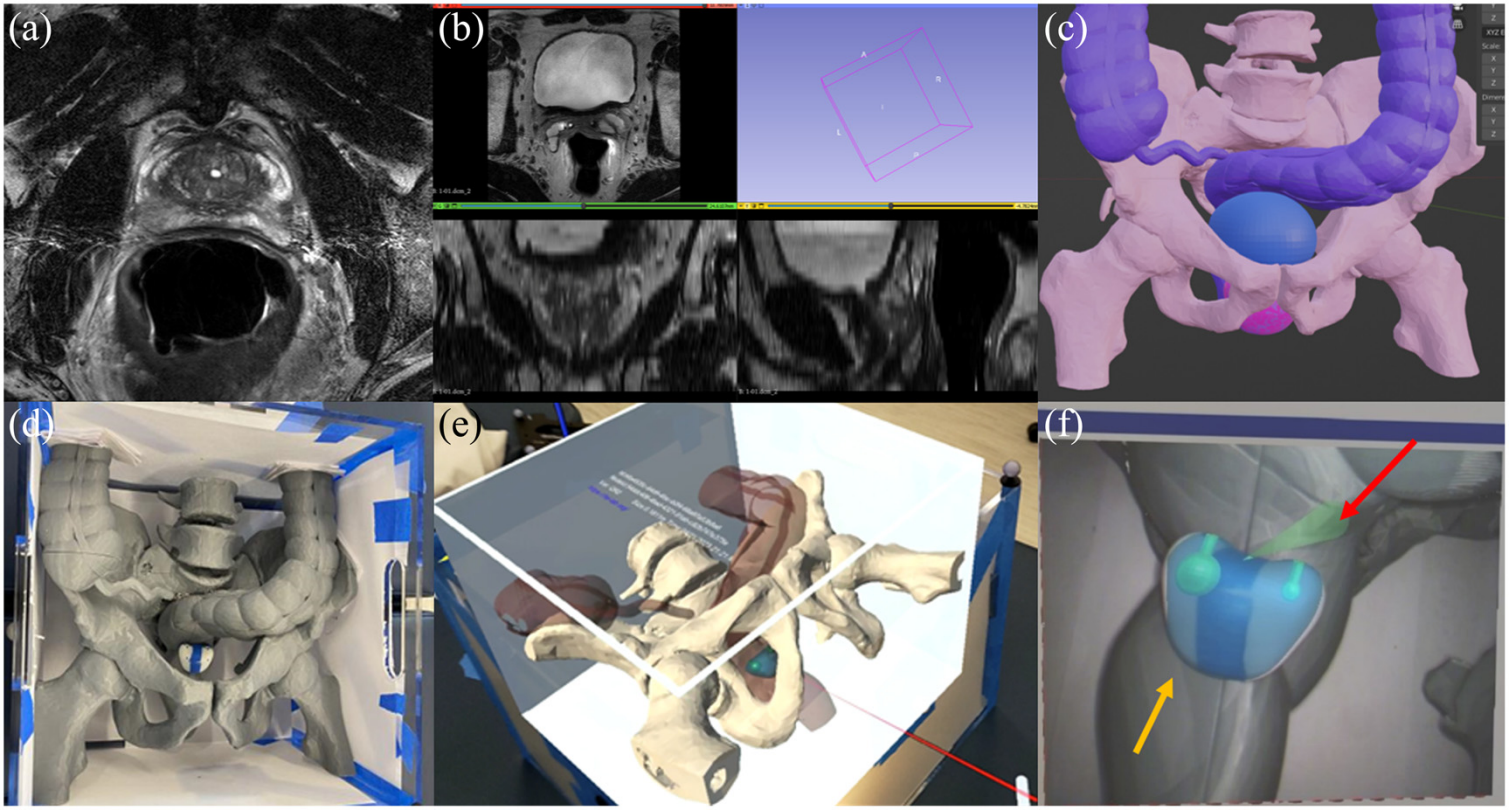
Workflow of the phantom design and 3D printing for the AR-guided procedures. (a) The MR image of a prostate and its surrounding organs. (b) Imported MR images in 3D Slicer for subsequent segmentation. (c) 3D model of the pelvic structures. (d) 3D-printed phantom for experiments. (e) AR view of the superimposed virtual model on the physical phantom. (f) The AR view of the prostate (yellow arrow) displayed on the real-time laparoscopic feed with the guidance beam (red arrow) displaying the target direction.

The AR system employs a publicly accessible MRI dataset^25^^,^^26^ to reconstruct virtual models of the patient’s internal anatomy, as illustrated in Figs. 2(a)–2(b).

During the interventional procedure, retroreflective markers enable real-time tracking of the patient’s position through the optical tracking system. This spatial information allows the system to accurately overlay virtual models of the identified anatomical structures onto their corresponding locations in the physical patient, providing the surgeon with AR-enhanced view that integrates critical anatomical details into both the camera feed and direct surgical field.

#### 2.1.2 Phantom model fabrication

To evaluate the performance of our AR-guided system, we created phantom models of the prostate and surrounding organs.^27^ The lesions were modeled as spheroidal structures with diameters of 5 and 10 mm, respectively. All components were scaled and positioned to accurately reflect the anatomical layout and dimensions of an adult male pelvic cavity of average size, as depicted in Fig. 2(c).

The 3D anatomical model from MRI was used to fabricate a physical phantom of identical dimensions using fused deposition modeling (FDM) 3D printing with polylactic acid (PLA). A rigid phantom was chosen to minimize discrepancies between the virtual and physical models, thereby reducing potential sources of measurement error. Deviations in size, shape, or geometry between the two representations could introduce systematic errors when assessing system accuracy. For this reason, the model design prioritized sufficient structural complexity over strict anatomical fidelity, particularly for organs less relevant to the evaluated procedure. As shown in Fig. 2(d), the phantom organs were housed in a plastic container equipped with optical markers for tracking. A virtual replica of the container was also rendered to complete the digital representation of the experimental setup. Virtual models were likewise created for all surgical instruments used in the study. Notably, the plastic container was originally transparent but was later rendered opaque using sheets of plain paper, as reflections and refractions from its translucent surface interfered with the infrared tracking system’s ability to accurately detect the marker positions.

#### 2.1.3 Implementation

**Fig. 3:**
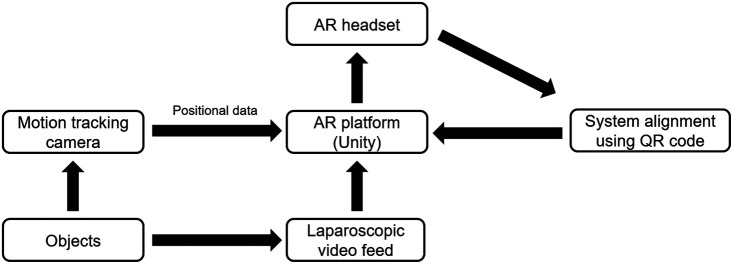
Pipeline of the AR platform for interventional procedures. To initialize the system, the user aligns the headset by focusing on a QR code, which serves as a reference point for synchronizing the coordinate origins of both the optical tracking system and the AR headset. The laparoscope, probe, and phantom models are each equipped with reflective markers for tracking. The optical tracking system continuously streams the six-degree-of-freedom poses of these tracked objects to Unity. The laparoscopic video feed is imported into Unity via OBS, where model registration is performed. MRTK is then utilized within Unity to enable AR visualization of the registered 3D model on the AR headset.

Figure 3 shows the interaction diagram among different AR sub-systems. Our motion tracking system performed localization of the phantom model and surgical tools and streamed tracking data to Unity via motion tracking software in real-time at 120 fps. This positional data was applied to the virtual models in Unity for location and orientation adjustment as well as hologram registration. When operators wear the AR headset, which is connected to Unity via MRTK, they can see virtual models overlaid onto their real-world counterparts as well as on a virtual window displaying the laparoscopic video feed as visualized in Figs. 2(e) and 2(f).

MRTK enables us to use a powerful computing system from the workstation instead of the on-board computing unit in the AR headset. A real-time laparoscopic video feed was captured using open broadcaster software (OBS). The video feed was streamed to Unity in real-time using a network device interface. The hologram registration was performed within Unity by adjusting the position of virtual representations until they were aligned with the phantom model in the video feed. Figure 2(f) displays the overlaid laparoscopic video feed. Moreover, A guidance beam is cast from the tip of the laparoscope toward the target and displays the direction of the target and whether the path is obstructed.

A QR code-based calibration method utilizing MRTK^28^ was implemented to establish a common coordinate frame between the Unity environment and the optical tracking system, thereby aligning the virtual phantom with its physical counterpart. Prior to calibrating the AR-guided system, the optical tracking system was initialized to define its global origin. The QR code was subsequently positioned at this origin, allowing the transformation between the QR code’s coordinate frame and the virtual object’s frame to be calculated as

$$T_{O}^{\mathrm{QR}}=T_{U}^{\mathrm{QR}}\cdot T_{O}^{U},$$ (1)

where T is the transformation, QR is the coordinate of QR code, O is the coordinate of a virtual object, and U is the coordinate system within the Unity engine.

### 2.2 AR-Guided System for Prostate Biopsy

#### 2.2.1 System development

**Fig. 4:**
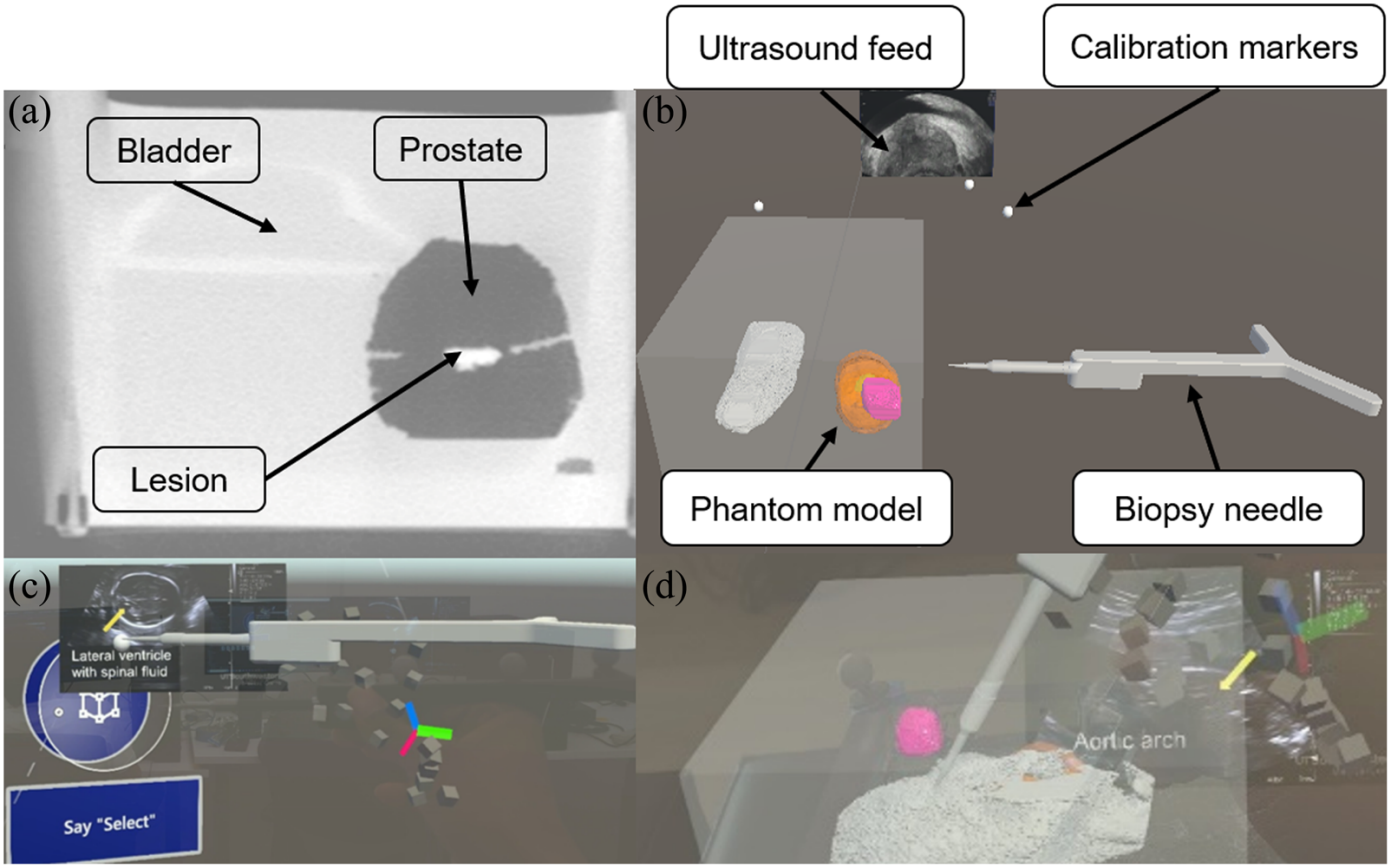
AR-guided system development pipeline for prostate biopsy. (a) The CT scan of phantom models. (b) Virtual models in Unity including real-time ultrasound feed. (c) The calibration process of the optical tracking system and Unity. (d) AR view of prostate biopsy using our proposed system.

Figure 4 shows the overall process of applying the AR-guided system for prostate biopsy. The motion tracking system was used to provide real-time tracking data of the target organ and surgical tools. However, we devised another calibration method to deal with the hologram visualization offset between the optical tracking system and Unity. Retroreflective markers were attached to the AR headset and the phantom model.

As shown in Fig. 4(a), CT images of the phantom model were acquired using a Ziehm Vision RFD 3D C-arm (Ziehm Imaging Inc., Orlando, Florida). The computer models of these phantoms were created using 3D Slicer software. Moreover, a virtual model of the probe was created for the sake of biopsy navigation.

Three calibration markers were created in Unity. The reason for using three markers is to ensure that there will not be depth inconsistencies during the calibration. Users need to align the probe kit with the calibration markers based on their viewpoint,^29^ as depicted in Fig. 4(b). The tracking data of physical objects was then streamed into Unity, and transformation between physical and virtual objects was calculated to minimize hologram visualization offsets between the optical tracking system and Unity as

$$T_{O}^{H}=T_{U}^{H}\cdot T_{O}^{U},$$ (2)

where T is the transformation, H is the coordinate of the AR headset, O is the coordinate of a virtual object, and U is the coordinate system within the Unity engine.

In addition, this AR-guided system provides real-time ultrasound feed in the user’s view, as depicted in Fig. 4(c), by importing it into Unity using OBS. This enables tracking of the biopsy needle inside the patient’s body. Figure 4(d) displays the AR view during the biopsy procedure using our AR-guided system.

#### 2.2.2 Phantom creation

**Fig. 5:**
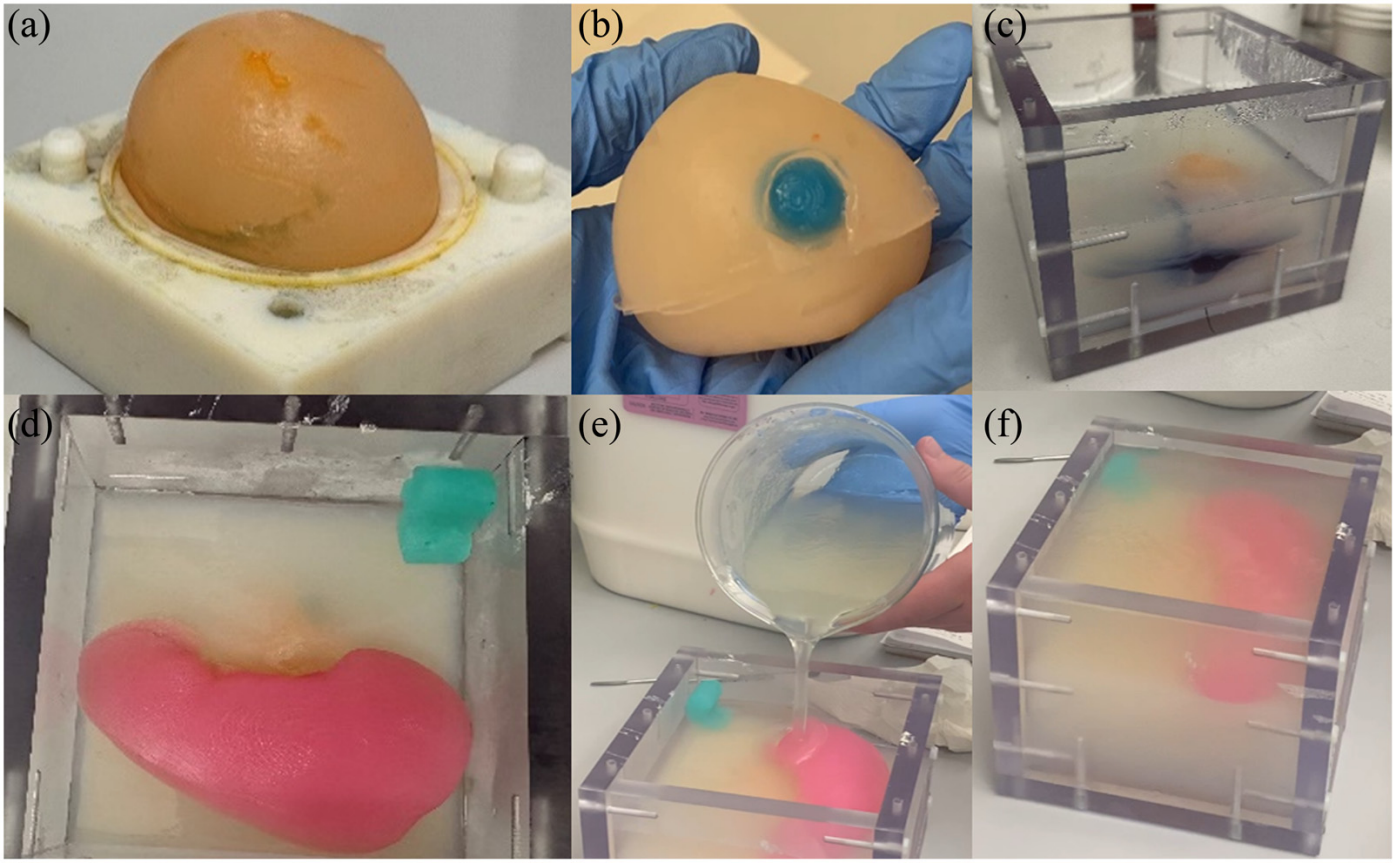
Fabrication process of the prostate phantoms. (a) The deformable prostate model. (b) The embedded lesion within the prostate model. (c) A phantom box with agar solution. (d) Phantoms of bladder (pink) and appendix (green). (e) Filling agar solution in the top layer. (f) The completed phantom model.

A deformable phantom model was used to evaluate our AR-guided system performance on prostate biopsies. The phantom model comprises a prostate with an embedded lesion, a bladder, an appendix, and the surrounding tissue. To create the phantom, a prostate mold was carved from Styrofoam and covered with agar, as seen in Fig. 5(a). The prostate model was cut in half to insert a lesion made from silicone-based putty. Both parts of the prostate model were glued back together as shown in Fig. 5(b). A heated solution of 2% w/v agar was poured into a plastic box as a base layer, and the prostate model was inserted as displayed in Fig. 5(c). Surrounding organs were created in a similar manner and included in the final model, as seen in Fig. 5(f).

### 2.3 Evaluation Metrics

Target registration error (TRE): For interventional procedures, TRE was utilized as the accuracy metric.^30^^,^^31^ This was measured using our high-precision optical tracking system. The TRE is defined as the Euclidean distance between the probe tip and target lesion upon collision of their virtual counterparts in the AR view. The TRE is calculated as

$$\mathrm{TRE}=\sqrt{{\left(\Delta P_{X}\right)}^{2}+{\left(\Delta P_{Y}\right)}^{2}+{\left(\Delta P_{Z}\right)}^{2}},$$ (3)

where $\Delta P_{x},\Delta P_{y},\Delta P_{z}$ are the distances between the probe tip and the target in the x, y, and z directions, respectively.

### 2.4 Evaluation of the AR Platform

Two potential applications in prostate interventions of the AR platform were explored. Two phantom experiments were conducted to evaluate the proposed AR platform. TRE was measured in both simulated laparoscopic and prostate biopsy procedures. To ensure experimental consistency and minimize inter-operator variability, a single operator was assigned to perform all trials for the laparoscopic application, whereas a separate operator conducted all trials for the biopsy application.

#### 2.4.1 Laparoscopic procedure

For TRE measurement, ten target points on the prostate model were manually defined around the lesion area using an ink marker. Spherical virtual markers were placed at their corresponding physical target locations.

**Fig. 6:**
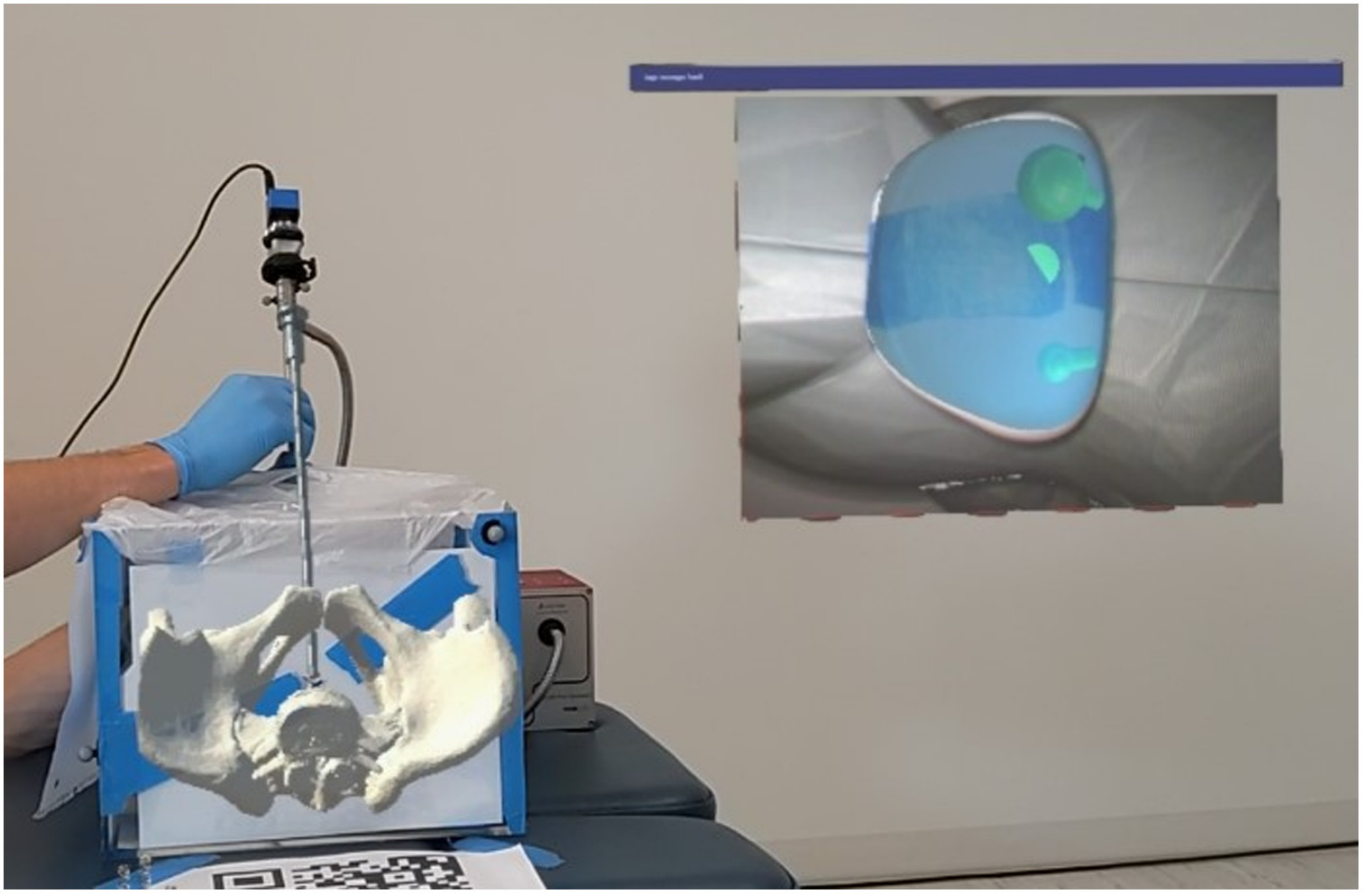
AR view of the operator during laparoscopic procedures. Virtual models of the phantom organs and the laparoscope are superimposed onto their physical counterparts. This additional visual information enables surgeons to localize target regions and surrounding organs more accurately, thereby improving targeting precision. The real-time laparoscopic video feed is displayed within the panel.

An operator then guided the surgical tool to each target point using the AR platform’s laparoscopic video feed. This procedure was repeated three times for each marker, and the TRE was subsequently calculated for each point relative to its corresponding virtual marker. Figure 6 illustrates an AR view of the operator during the experiment.

#### 2.4.2 Prostate biopsy applications

**Fig. 7:**
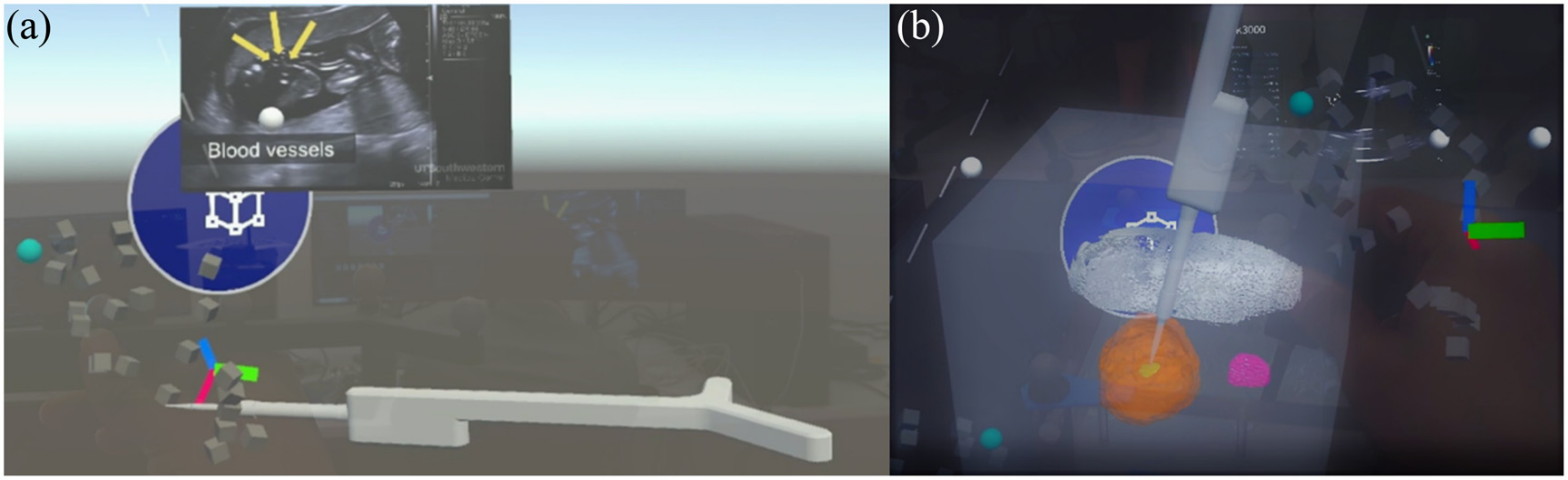
Examples of AR view during the biopsy procedure. (a) The calibrated probe within the user’s hand. (b) The prostate cancer (yellow object) sample was collected using the probe.

The intra-prostatic lesion within the deformable phantom was designated as the target. TRE was calculated as the Euclidean distance between the probe tip and the lesion’s margin. If the probe tip is located within or on the boundary of the lesion, a TRE of zero is recorded. To facilitate tracking, optical markers were affixed to each corner of the phantom housing. Prior to the trials, the probe was calibrated as illustrated in Fig. 7(a).

Prostate biopsies were simulated by piercing the probe through the intra-prostatic lesion in the phantom, as depicted in Fig. 7(b). In addition, the real-time ultrasound feed was streamed in Unity and visualized in the operator’s field of view to facilitate the procedure.

## 3 Results

The performance of the high-speed optical tracking AR-guided platform was evaluated by quantifying the TRE. We conducted four sets of experiments, comprising trials for both laparoscopic and biopsy procedures alongside their respective baseline evaluations.

### 3.1 AR-Guided Laparoscopic Procedure

**Fig. 8:**
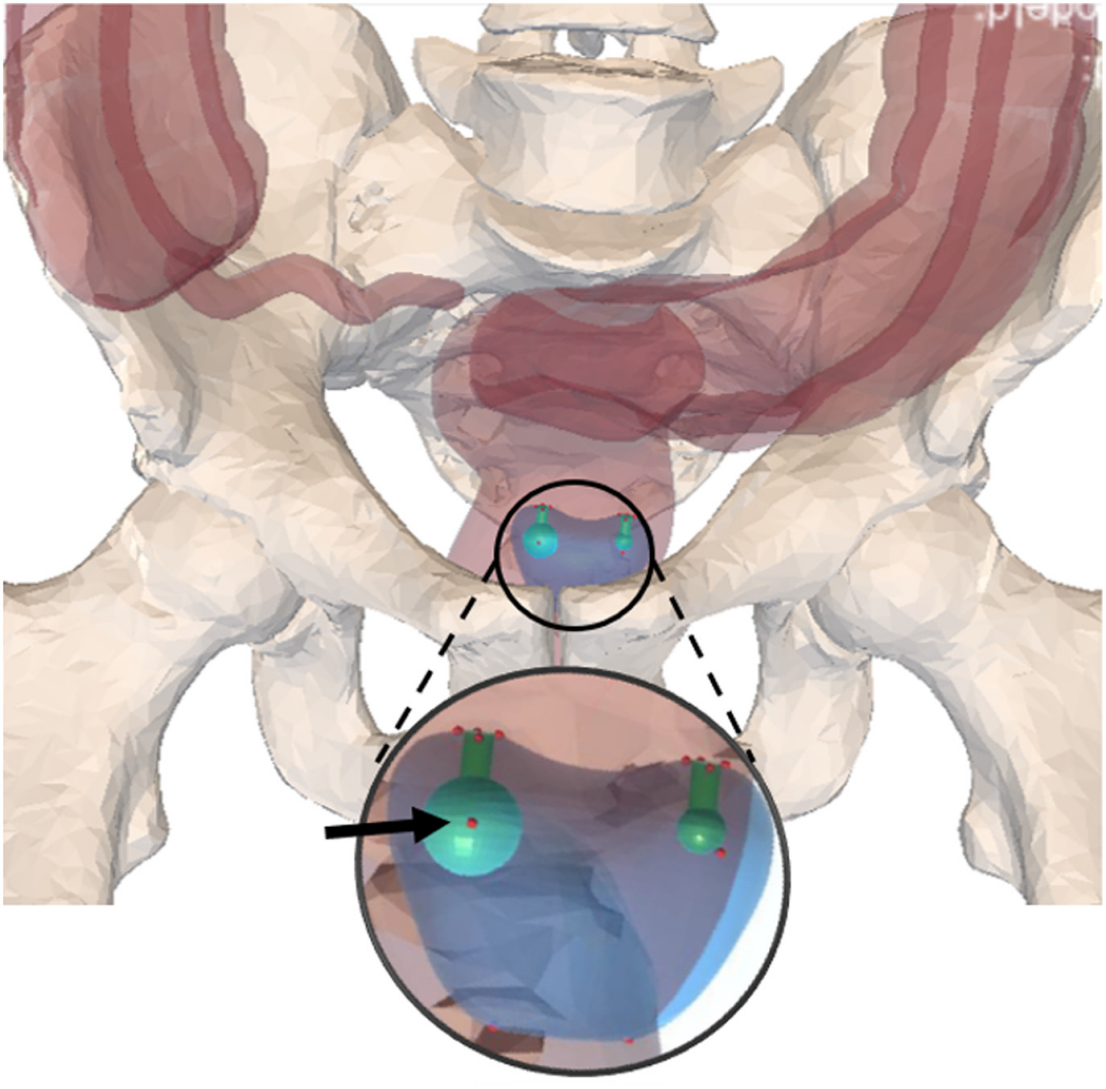
Virtual model of the physical phantom with predefined virtual markers (red dot as shown by the arrow) for the targeting experiment within the real-time laparoscopic video feed.

**Fig. 9:**
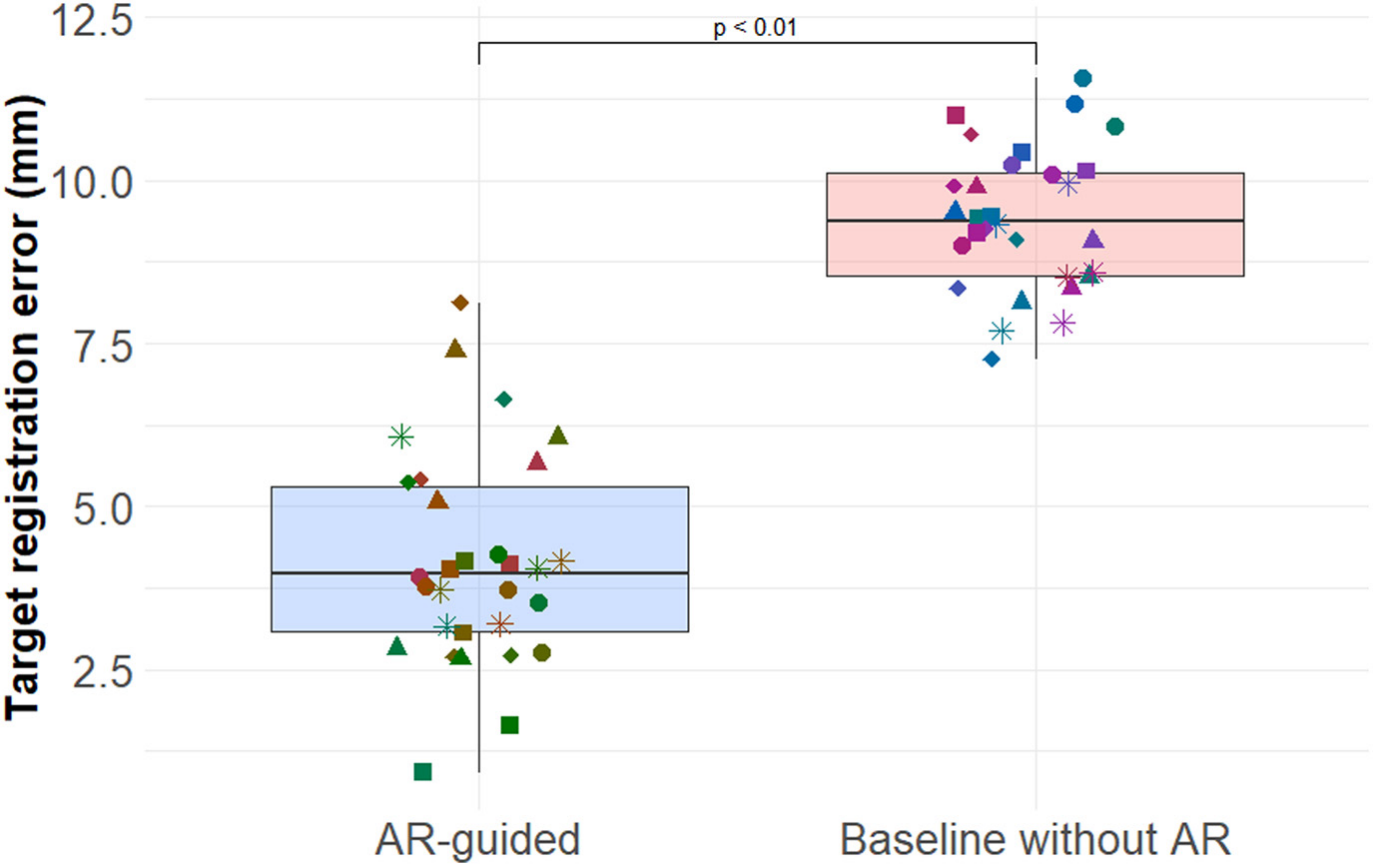
Box-and-whisker plot of the target registration errors of the AR-guided laparoscopic procedure and its baseline without AR guidance.

Figure 8 shows the location of virtual markers within the virtual model of the phantom for TRE evaluation. The procedure of this experiment is based on Sec. 2.4.1. Figure 9 shows that the AR-guided approach significantly improved accuracy compared with the baseline. Across three trials of simulated laparoscopic surgery, with an average TRE of 4.17±1.63  mm for all markers and trials, whereas the baseline method yielded a mean TRE of 9.42±1.09  mm (Student’s t-test, p<0.01).

### 3.2 AR-Guided Prostate Biopsy

**Fig. 10:**
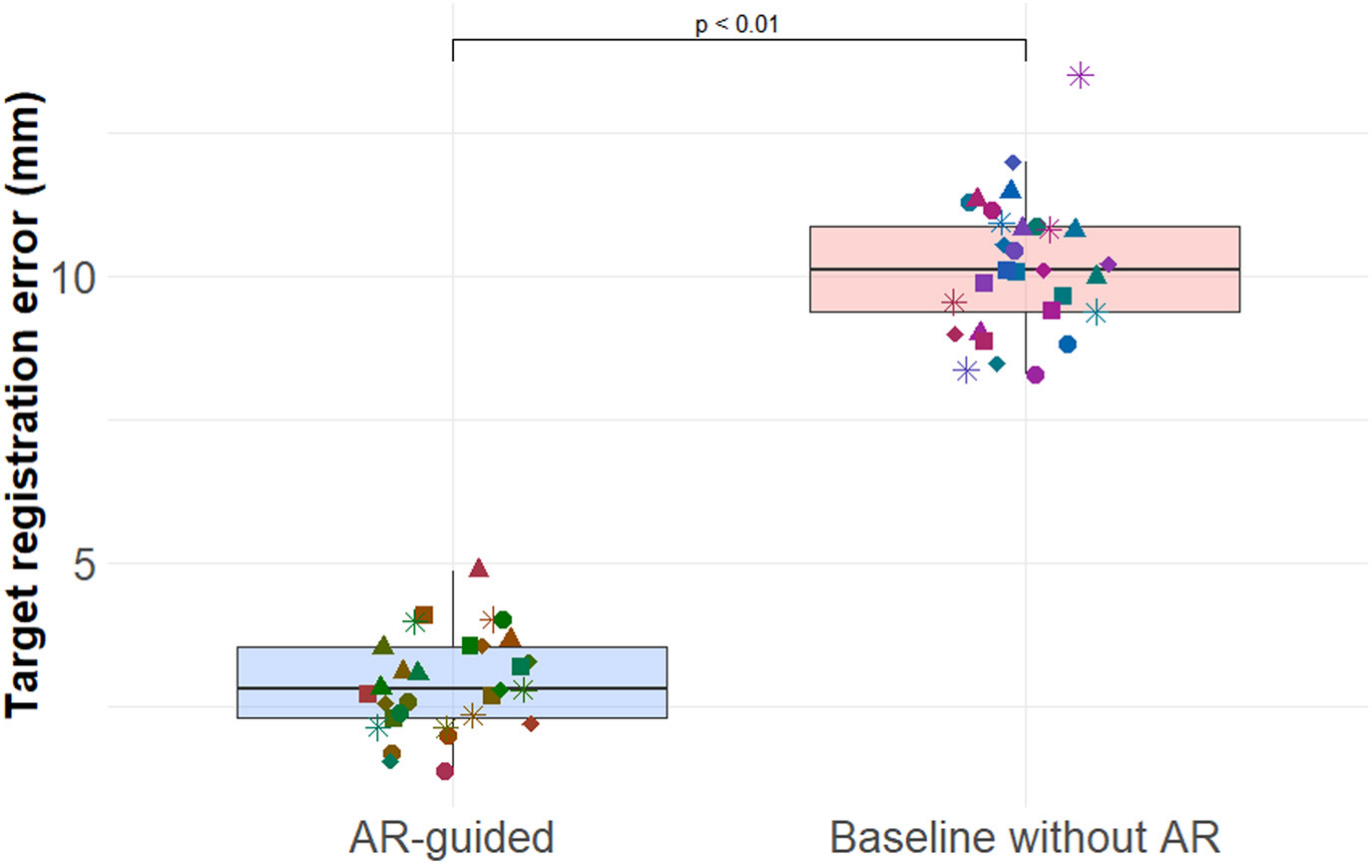
Box-and-whisker plots of the target registration errors of the AR-guided biopsy procedure and its baseline without AR guidance.

The procedure for AR-guided biopsy evaluation experiment was described on Sec. 2.4.2. The average TRE for the 30 trials of the simulated prostate biopsy was 2.89±0.84  mm. In comparison, the baseline experiment yielded an average TRE of 10.17±1.18  mm. This represents a statistically significant difference compared with the AR-guided procedure (Student’s t-test, p<0.01), as illustrated in Fig. 10.

## 4 Discussion and Conclusion

We developed a high-speed optical tracking and AR platform for interventional procedures. We also applied the system for laparoscopic procedures and prostate biopsies on phantoms. The AR-guided system achieved TRE at the millimeter level. The AR platform provides an anatomical overlay of the target and surrounding organs using 3D virtual models. Precise hologram registration of the organ of interest was achieved by integrating an optical tracking system into our AR platform.

Related works by Li et al.^32^ and Lin et al.^33^ similarly utilized both an AR headset and an optical tracking system as primary components. Li et al.^32^ developed an AR-guided system for transperineal free-hand prostate procedures, where reference frames for both the patient and needle were established using fiducial markers. Their approach employed a custom application to perform point-to-point registration between virtual organ models and the phantom. In contrast, our proposed method utilizes a QR code placed adjacent to the patient, thereby minimizing interference with the operator during the procedure. Similarly, Lin *et al.*^33^ leveraged positional data from the AR headset, phantom, and biopsy needle, acquired via optical tracking, to guide needle placement. Distinct from this approach, our AR-guided system integrates this positional data within the Unity platform to visualize both virtual models and real-time ultrasound feeds simultaneously.

The high-speed optical tracking and AR platform has potential applications in prostate biopsy and minimally invasive surgery. Our TRE is similar to the work by Tan et al.^34^ and Li et al.^32^ Lin et al.^33^ developed an AR-guided system for needle placement called HoloNeedle. They achieved a mean TRE of 8.15±0.4  mm for needle placement when applying rigid body registration. Nevertheless, we can achieve a smaller mean TRE of 4.17 mm, as demonstrated in this study.

Some limitations of our AR platform include the need for initial manual hologram registration. To further improve our AR platform, we plan to develop a new automated pipeline from image segmentation to hologram registration. Yu et al.^35^ illustrated an automatic tissue surface reconstruction technique using deep learning-based visual simultaneous localization and mapping (vSLAM) to create virtual models of target organs. The quality of a virtual model can be improved by integrating estimated depth maps into the surface reconstruction workflow.^36^ Furthermore, the impact of perceptual factors on the proposed AR-guided platform needs to be explored. For example, future studies should investigate how the blending of real and virtual objects affects positional accuracy and how different rendering styles might improve the overall precision of the platform.

In addition, abdominopelvic organs undergo motion and deformation due to respiration and surgical manipulation, which can lead to hologram registration errors during operation. Deformable registration algorithms should be investigated and implemented into the platform to provide more accurate hologram registration when used with soft tissue. The high-speed tracking and AR platform is robust across different interventional procedures and can be extended to other surgical applications.

## Data Availability

Code and data underlying the results presented in this paper are not publicly available at this time but may be obtained from the authors upon reasonable request.
